# Unilateral versus bilateral pedicle screw fixation for treating two-level lumbar degenerative diseases

**DOI:** 10.1038/s41598-025-13015-1

**Published:** 2025-07-27

**Authors:** Ke Yang, Dongwei Wu, Jianjun Kong, Xiangping Peng, Huarong Wu, Shaofeng Wang, Huiwang Wang, Xingtong Zhu, Zhanyong Wu, Sidong Yang

**Affiliations:** 1 Department of Orthopedic Surgery, Orthopaedic Hospital of Xingtai, Orthopaedic Research Institute of Xingtai, 202 Bayi Street, Xingtai, 054000 China; 2 Department of Orthopedic Surgery, Hebei Medical University Third Hospital, Shijiazhuang, 050051 China; 3 North China University of Science and Technology College of Nursing and Rehabilitation, Tangshan, 063000 China

**Keywords:** Unilateral pedicle screw fixation, Lumbar degenerative disease, Intervertebral fusion, Posterolateral fusion, Adjacent segment degeneration, Screw loosening, Diseases, Neurological disorders, Neurodegeneration, Neurodegenerative diseases

## Abstract

This study aimed to compare the clinical efficacy and radiological results between unilateral pedicle screw fixation (UPSF) and bilateral pedicle screw fixation (BPSF) for treating two-level lumbar degenerative diseases. The study involved 106 patients with two-level lumbar degenerative diseases. In the UPSF group (*n* = 52), 28 underwent intervertebral fusion (IF), 17 underwent posterolateral fusion (PLF), and 7 underwent IF & PLF. In the BPSF group (*n* = 54), 43 underwent IF, 2 underwent PLF, and 9 underwent IF & PLF. The clinical efficacy and radiological data were evaluated and compared. The UPSF group was significantly lower than the BPSF group regarding operation time, intraoperative blood loss, and hospitalization expenses. Postoperative visual analog scale scores and oswestry disability index were significantly decreased in both groups compared with pre-surgery. At the last follow-up, the intervertebral disc height of the cranial adjacent segment decreased more in the UPSF group compared with the BPSF group. The incidence of screw loosening in the UPSF group was significantly higher compared with the BPSF group. Compared to BPSF, UPSF can achieve similar and satisfactory clinical efficacy for the treatment of two-level lumbar degenerative diseases with less operation time, muscle damage, blood loss, and hospitalization expenses. However, the occurrence of pedicle screw loosening was higher in the UPSF group.

## Introduction

In clinical scenarios, intervertebral disc diseases are mainly caused by intervertebral disc degeneration, which can result in lower back pain and leg pain^1–3^. For lumbar degenerative diseases, pedicle screw fixation and interbody fusion are classical and standardized surgical approaches. The efficacy of this intervention has been demonstrated in terms of spinal stability reconstruction, restoration of intervertebral disc and foramina height, decompression of nerve roots, and subsequent relief of clinical symptoms^4^. The bilateral pedicle screw fixation (BPSF) significantly contributes to the local stability maintenance of the surgical segments. However, it also leads to an increased mechanical load on the adjacent discs and facet joints, thereby accelerating the progression of adjacent segment degeneration (ASD)^5,6^. The literature has reported that radiographic and clinical ASD rates after lumbar spine fusion range from 5 to 77% and 0–27%, respectively^6^. Bone mineral of the fixed segments can decrease due to the effect of stress shielding^7^. Therefore, Kabin et al.^8^ first proposed unilateral pedicle screw fixation (UPSF) for the treatment of L4-5 lumbar degenerative diseases in 1992 and achieved similar clinical effects to BPSF. Compared with the BPSF group, the UPSF group reduces the strength of pedicle screw fixation and the incidence of ASD and has advantages of less muscle injury, operation time and hospitalization cost^9–12^.

Minimally invasive technique is an inevitable trend in the development of spine surgery. Since Kabin et al.^8^ proposed the concept of UPSF for the treatment of lumbar degenerative diseases, this approach has been often used in clinical scenarios because of its advantages. Currently, most of the reports are about the single-level UPSF and two-level UPSF are not many^13–16^. Mao et al.^17^ found in the clinical study that UPSF for the treatment of two-level lumbar degenerative diseases can effectively improve the lordosis of the whole lumbar spine and the surgical segments. Suk et al.^18^ performed UPSF and combined it with posterolateral fusion (PLF) to treat two-level lumbar degenerative diseases and gained satisfactory clinical efficacy. In recent years, only a few studies have reported the use of UPSF for treating two-level lumbar disc diseases^15,19–21^. The main controversy is that the UPSF for two-level segments may cause the risk of poor biomechanical stability^22^. However, the protective factors of the fixation, such as muscle and ligament, were not considered in the biomechanical tests^23^. In addition, these previous studies did not assess the ASD and screw loosening related to UPSF or BPSF. Further studies are needed to compare UPSF with BPSF in treating two-level lumbar disc diseases^17^. Thus, BPSF and UPSF were compared for two-level lumbar degenerative diseases on the basis of clinical effectiveness and radiological results.

## Methods

### Ethics statement

This retrospective study was approved by the Ethics Committee of the Orthopaedic Hospital of Xingtai (approval No. ZCKT-2025-0004). All procedures were performed in accordance with the relevant guidelines and regulations of the Declaration of Helsinki. We confirm that all participants have obtained informed consent. And each patient was informed of treatment-related risk factors and complications, treatment options before surgery, and signed a written informed consent.

### Inclusion and exclusion criteria

Inclusion criteria: (1) lumbar degenerative diseases, containing lumbar disc herniation (LDH) and lumbar spinal stenosis (LSS), at two consecutive levels, including L3-L5 or L4-S1; (2) Mild lumbar spondylolisthesis of one segment (Meyerding grade I) concurrent with LSS or LDH in adjacent segments; (3) typical low back pain and lower limb symptoms; (4) The radiological results were consistent with the symptoms. The symptoms caused by the above degenerative lumbar diseases lasted for more than 6 months and did not relieve after conservative treatment such as physical and drug therapy.

Exclusion criteria: (1) the degree of lumbar degenerative spondylolisthesis is equal or greater than II; (2) Incomplete follow-up data; (3) A history of lumbar L3-5 and L4-S1 surgery; (4) lumbar vertebra fracture, spinal deformity, infection, tumor, severe osteoporosis, serious smoking history and excessive obesity;

### Surgical techniques

UPSF Group: Under general anesthesia, the patient was placed in a prone position on a radiolucent operating table. Afterwards, a longitudinal 8–10 cm skin incision was made at the surgical area on the lower back. The skin, subcutaneous tissue, and fascia were meticulously dissected layer by layer. The unilateral paravertebral muscles, on the symptomatic side or more severe pathological side, were split up to the outer edge of the facet process. And the pedicle screws were carefully inserted (ensure the protection of the complete structure of the superior articular capsules) while preserving the integrity of the posterior spinous ligament complex and contralateral facet structure. The medial portion of the inferior articular process was excised (if interbody fusion was performed to remove the entire inferior articular process), and sections of the upper, lower laminae and the ligamentum flavum were dissected. The nerve root was carefully manipulated and protected under direct visualization, ensuring complete decompression and release. The nucleus pulposus was excised, while complete removal of the upper and lower intervertebral cartilage endplates was performed. In the case of contralateral compression, contralateral covert decompression of nerve roots was performed. The spinous process was fractured using a bone knife to expose the space between the central ligamentum flavum and the dural sac. Nerve strippers were used to separate this space, allowing for depression of the dural sac. Gradual removal of the contralateral ligamentum flavum was carried out to enhance the exposure of the contralateral space (if necessary, a microscope can be utilized, and the operating table rotated approximately 20°towards the contralateral side). This approach facilitated the release and decompression of both the contralateral spinal canal and nerve roots. After these procedures, intraoperative spinal canal angiography was conducted to assess the efficacy of decompression. Following the implantation of partial autogenous bone in the intervertebral space (autogenous iliac bone can be used if there is insufficient bone graft), a fusion device filled with bone fragments was inserted into the intervertebral space (with an oblique placement ensuring that the front end of the fusion device reached as far as possible towards the opposite side^21^. The same procedure was performed to another intervertebral disc. C-arm fluoroscopy confirmed satisfactory positioning of both the interbody fusion apparatus and screws, which were subsequently tightened securely. In cases where posterolateral bone grafting was required, bone knives were utilized to prepare a suitable graft bed within either the articular process space or between transverse processes. Decompressed bone fragments were then implanted, drainage tubes were placed, and finally, layer-by-layer closure of the incision was conducted.

BPSF Group: Standard transforaminal lumbar interbody fusion (TLIF) involved a posterior median incision, bilateral muscle dissection, insertion of bilateral pedicle screws, retention of the posterior ligament complex, and decompression based on preoperative symptoms and imaging data. Other procedures used were consistent with those in the UPSF group.

### Postoperative treatment

Antibiotics were administered intravenously within 24 h post-surgery as a prophylactic measurement against potential infection. Close monitoring of lower-limb activity was conducted, and the patient was instructed to perform appropriate exercises in bed to prevent thrombosis. The drainage was removed between 48 and 72 h after surgery depending on the amount of fluid inside (< 50 ml in 24 h). Following deep venous ultrasonography of both lower limbs, a waist brace was worn to help support the lumbar spine for three months.

### Clinical efficacy and radiological evaluation

The following radiological images were performed: lateral, anterior-posterior and flexion-extension lumbar spine radiographs, CT, and MRI scans before surgery; lateral and anterior-posterior lumbar spine radiographs and CT scans at 3 months and the final follow-up post-surgery. The operation duration, intraoperative blood loss, incidence of postoperative complications, hospitalization costs, and hospital stays of postoperative were recorded for both groups. The Oswestry Disability Index (ODI) and Visual Analog Scale (VAS) scores for low back pain and leg pain were assessed preoperatively, at 3 months post-surgery, and at the final follow-up.

**Fig. 1:**
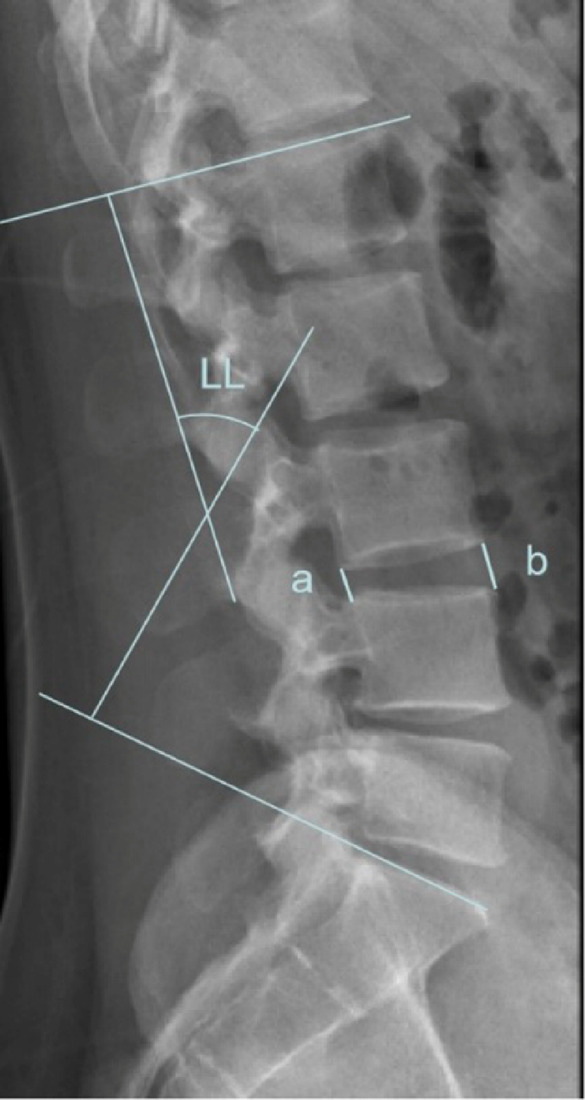
Methods of measuring lordosis (LL) angle and intervertebral disc height (IDH). The measurement method for LL angle involved drawing parallel lines on L1 endplate and S1 endplate from lateral lumbar spine X-rays, followed by drawing two perpendicular lines to determine the angle. IDH was measured by averaging the distances between anterior and posterior edges of vertebral bodies on lateral lumbar X-rays (IDH= (a + b)/2).

The LL angle and IDH in the proximal segment of the head were measured preoperatively, at 3 months, and during the final follow-up. Meanwhile, during the X-ray examination at the final follow-up, we measured the 1 mm radiolucent band surrounding the implant to assess the stability of the screws. The measurement method for the LL angle involved drawing parallel lines on the L1 endplate and S1 endplate from lateral lumbar spine X-rays, followed by drawing two perpendicular lines to determine the angle. IDH was measured by averaging the distances between anterior and posterior edges of vertebral bodies on lateral lumbar X-rays (IDH= (a + b)/2, Fig. 1). CT evaluation based on Bridwell score was performed during the final follow-up to assess IF or PLF. The evaluation criteria were as follows: Grade I: Fusion with bone remodeling and trabecular formation. Grade II: Complete bone graft but incomplete bone remodeling without clear areas. Grade III: Intact graft with clear areas at upper and lower ends. Grade IV: Non-fusion with graft resorption leading to collapse. To ensure the accuracy and reliability of the determination of screw loosening, in this study, two senior radiologists were invited to independently evaluate all imaging materials to determine whether the screws were loose.

### Statistical analysis

Data analysis was performed using SPSS 24.0 statistical software (SPSS, Inc., Chicago, IL, USA). The measurement data were presented as mean ± standard deviation (SD). VAS score and ODI were presented as median (Interquartile Range, IQR). The gender composition, surgical segment distribution, preoperative diagnosis and fusion rate of the two groups were compared using either Chi-square tests or Fisher’s exact tests. Age, BMI, follow-up time, operation time, intraoperative blood loss, hospitalization cost, and postoperative hospital stays were analyzed using independent samples t-tests. The comparisons of VAS score and ODI were performed using nonparametric rank-sum tests. The comparisons of radiological parameters at each follow-up time point pre- and post-surgery were conducted using repeated measures Analysis of Variance (ANOVA) followed by post-hoc LSD-t tests for multiple comparisons. Statistical significance was set at *P* < 0.05.

## Results

### Baseline characteristics

A total of 106 patients with two-level lumbar degenerative diseases who visited our hospital between January 2015 and May 2020 were included in this study (Table 1). There were 52 patients in the UPSF group, including 27 males and 25 females. There were 42 cases of LDH or LSS, and 10 cases with lumbar spondylolisthesis. For operative segments, there were 19 cases of L3–L5 and 33 cases of L4-S1. As for different fusion techniques, there were 28 cases of IF, 17 cases of PLF, and 7 cases of IF & PLF. In the BPSF group, there were 54 cases, including 28 males and 26 females. There were 41 cases of LDH or LSS, and 13 cases with lumbar spondylolisthesis. For the operative segment, there were 23 cases of L3–L5 and 31 cases of L4-S1. For fusion techniques, there were 43 cases of IF, 2 cases of PLF, and 9 cases of IF & PLF. There was no significant difference between the two groups regarding Age (*P* = 0.936), gender (*P* = 0.854), BMI (*P* = 0.106), follow-up period (*P* = 0.357), preoperative diagnosis (*P* = 0.925), and operative segments (*P* = 0.250) (Table 1).

**Table 1:** Baseline characteristics of patients in two groups.

	BPSF Group (*n* = 54)	UPSF Group (*n* = 52)	t/χ^2^ value	*P* value
Age (years)	52.03 ± 12.09	52.25 ± 15.20	0.080	0.936
Gender (male/female)	28/26	27/25	0.034	0.854
BMI	26.26 ± 2.79	27.08 ± 2.34	1.631	0.106
Follow-up period (months)	23.07 ± 11.15	25.04 ± 10.70	0.925	0.357
*Preoperative diagnosis*			0.009	0.925
LSS/ LDH	41	42		
Combined with spondylolisthesis	13	10		
Operative segment			1.322	0.250
L3–L5	23	19		
L4–S1	31	33		

*UPSF* unilateral pedicle screw fixation, *BPSF* bilateral pedicle screw fixation, *BMI* body mass index, *LDH* lumbar disc herniation, *LSS* lumbar spinal stenosis.

### Perioperative results and screw loosening

**Fig. 2:**
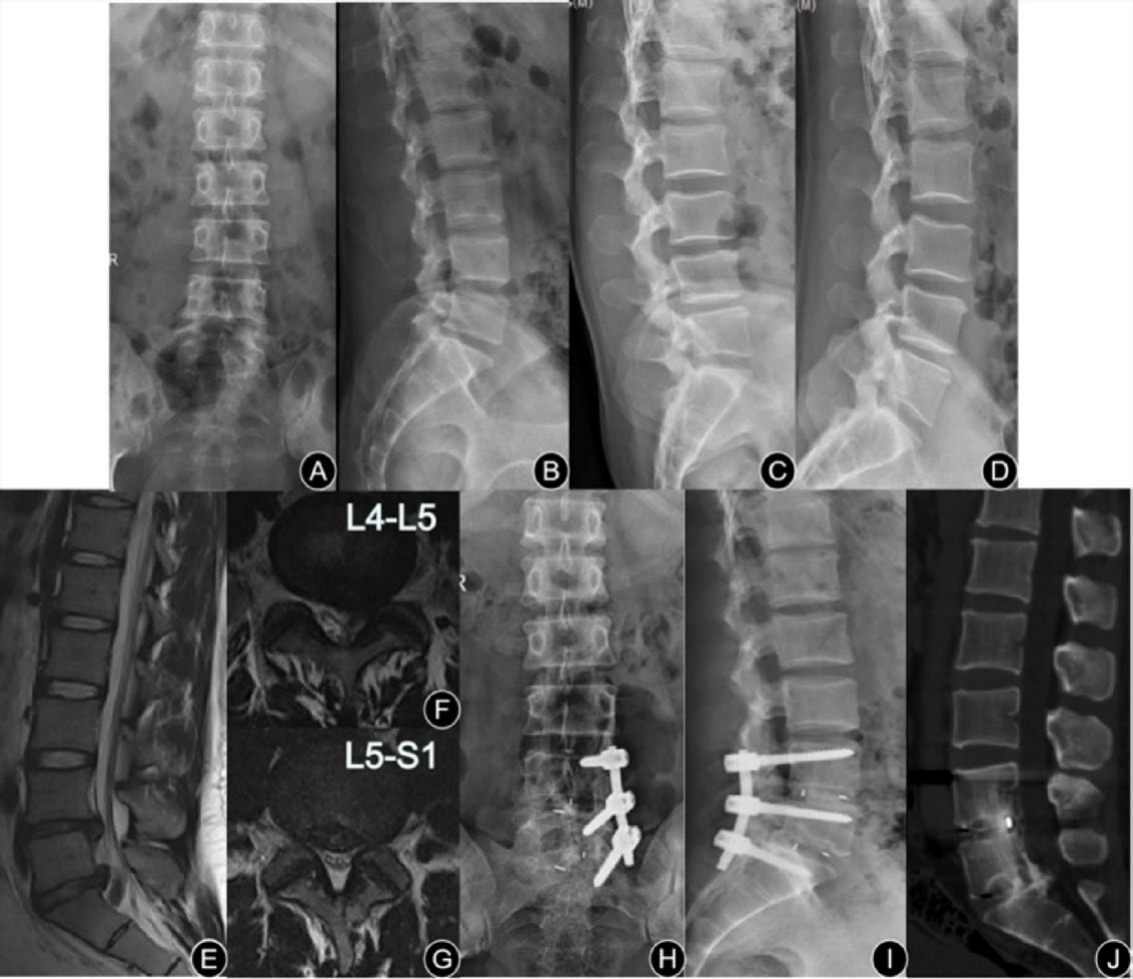
Unilateral pedicle screw fixation (UPSF) combined with intervertebral fusion (IF) to treat two-level lumbar degenerative disease. Male, 39 years old, complaining with lower back pain and radiating pain, numbness of both legs for half a year, and the left is more serious. Preoperative diagnosis was L4-S1 lumbar disc herniation (LDH). (**A**)–(**D**) Anteroposterior, lateral, extension and flexion position plain indicated no instability and olisthy. (**E**) and (**G**) Preoperative MRI showed L4-S1 LDH; (**H**) and (**I**) Anteroposterior and lateral radiograph at 4 years after-surgery indicated that the location of internal fixation was good; (**J**) CT at 4 years after-surgery showed IF was excellent.

**Fig. 3:**
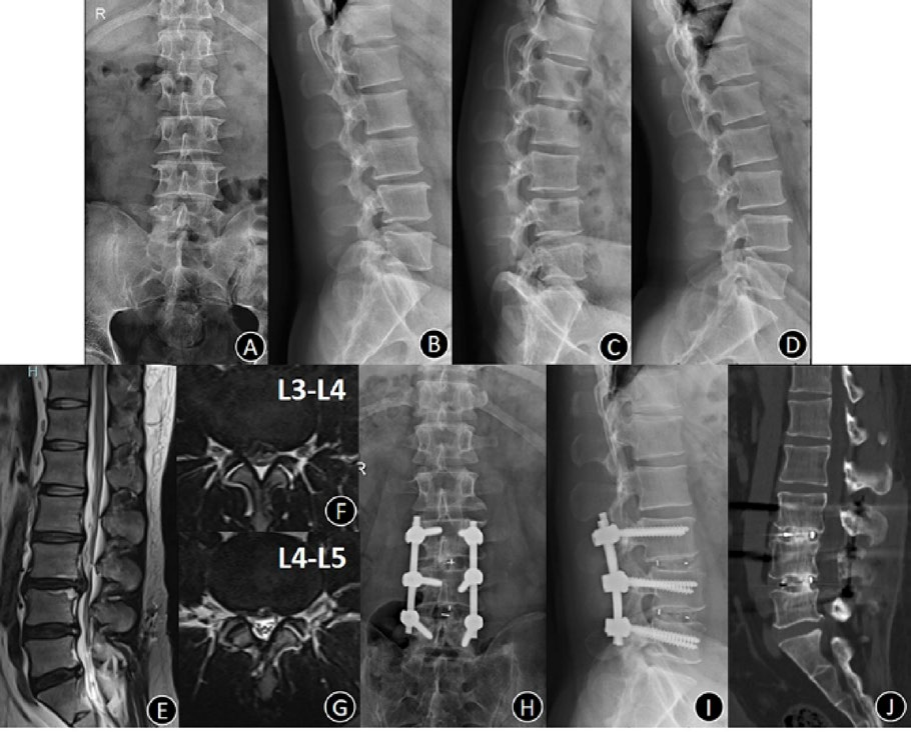
Bilateral pedicle screw fixation (BPSF) combined with intervertebral fusion (IF) to treat two-level lumbar degenerative disease. Male, 33 years old, complaining with lower back pain and radiating pain of left leg for two years. Preoperative diagnosis was L3-L5 lumbar disc herniation (LDH). (**A**)–(**D**) Anteroposterior, lateral, extension and flexion position plain indicated no instability and olisthy. (**E**) and (**G**) Preoperative MRI showed L3-L5 LDH; (**H**) and (**I**) Anteroposterior and lateral radiograph at 2 years after-surgery indicated that the location of internal fixation was good; (**J**) CT at 2 years after-surgery showed IF was excellent.

**Fig. 4:**
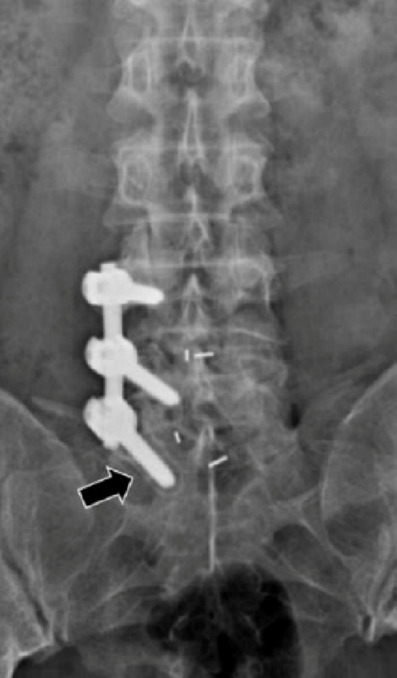
The screw loosening was occurred in surgery of unilateral pedicle screw fixation (UPSF) combined with interbody fusion (IF). Male, 59 years old, preoperative diagnosis was L4-S1 lumbar spinal stenosis (LSS). Anteroposterior radiographs of the follow-up 4 years after the surgery indicates screw loosening at caudal side (radiolucent line around the implant > 1 mm wide).

The surgical procedures were successfully performed to all patients. (Typical cases, Figs. 2 and 3). The operative time of the UPSF group was 131.75 ± 23.64 min, which was significantly shorter than the BPSF group 195.18 ± 45.81 min (*P* < 0.001). The intraoperative blood loss in the UPSF group was 329.42 ± 173.9 ml, significantly less than that in the BPSF group 643.15 ± 117.91 ml (*P* = 0.010). The hospitalization cost in the UPSF group was CNY 34546.09 ± 7781.32, significantly less than that in the BPSF group CNY 45404.34 ± 4721.55 (*P* < 0.001). The UPSF group demonstrated significant advantages in terms of operation time, intraoperative blood loss, and hospitalization costs. The postoperative hospital stays in the UPSF group 8.71 ± 3.36 days were significantly shorter than the BPSF group 10.51 ± 3.12 days (*P* = 0.005). Screw loosening occurred in 20 cases (Fig. 4) within the UPSF group and 7 cases within the BPSF group at the last follow-up after surgery. The incidence of screw loosening was significantly higher in the UPSF group compared to the BPSF group (*P* = 0.003, Table 2). The evaluation results of the two radiologists on whether the screws were loose showed good consistency. Intraclass Correlation Coefficient (ICC) analysis indicated that the ICC value was 0.79 (95% confidence interval: 0.78–0.91), with *P* < 0.001, suggesting that the two evaluators had good consistency in judging the loosening of the screws.

**Table 2:** Comparison of perioperative indicators, complications, and fusion rates between the two groups.

	BPSF Group (*n* = 54)	UPSF Group (*n* = 52)	t/χ^2^ value	*P* value
Operation duration (min)	195.18 ± 45.81	131.75 ± 23.64	– 9.032	*< 0.001*
Intraoperative blood loss (ml)	643.15 ± 117.91	329.42 ± 173.9	– 10.922	*0.010*
Hospitalization costs (yuan)	45404.34 ± 4721.55	34546.09 ± 7781.32	– 8.722	*< 0.001*
Hospital stays of postoperative (day)	10.51 ± 3.12	8.71 ± 3.36	– 2.867	*0.005*
*Postoperative complications*				
Dural tear	2	1		
Epidural hematoma	0	1		
Screw loosening	7/54	20/52	9.073	*0.003*

*UPSF* unilateral pedicle screw fixation, *BPSF* bilateral pedicle screw fixation.

### Fusion rate

According to the radiological results of the latest follow-up, the patients in the UPSF and BPSF groups were divided into three groups based on different fusion methods: IF group, PLF group, and IF & PLF group. In the UPSF group: the fusion rate was 96.43% in the IF group, with 25 cases grade I, 2 cases grade II, and 1 case grade III. The fusion rate was 82.35% in the PLF group, including 13 cases grade I, 1 case grade II, and 3 cases grade III. The IF & PLF group achieved a complete fusion rate of 100%, consisting of six cases grade I and one case grade II. In the BPSF group: a high overall fusion rate of 97.67% at the IF group, encompassing 35 cases grade I, 7 cases grade II, and 1 case grade III. The PLF group exhibited a perfect fusion in all cases (100%), with 1 case grade I and another case grade II; no cases were classified grade III in this subgroup. Furthermore, the IF & PLF group achieved a 100% fusion rate. This subgroup consisted of 7 cases grade I, 2 cases grade II, and no grade III. Importantly, the statistical analysis revealed that there were no significant differences among all groups regarding their respective fusion rates (*P* = 0.337, Table 3).

**Table 3:** Comparison of fusion rate of different fusion methods between two groups.

Group	Bridwell			Fusion rate
	Grade I	Grade II	Grade III	
*UPSF*				
IF	25	2	1	96.43%
PLF	13	1	3	82.35%
IF & PLF	6	1	0	100%
*BPSF*				
IF	35	7	1	97.67%
PLF	1	1	0	100%
IF & PLF	7	2	0	100%
χ^2^ value	–	–	–	0.921
*P* value	–	–	–	0.337

*UPSF* unilateral pedicle screw fixation, *BPSF* bilateral pedicle screw fixation, *IF* intervertebral fusion, *PLF* posterolateral fusion.

### Clinical efficacy

The analysis of the postoperative rehabilitation index revealed that the VAS leg scores in the UPSF group were 6 (IQR = 1) preoperatively, which significantly improved after surgery with a final follow-up score of 1 (IQR = 1). Similarly, in the BPSF group, the preoperative VAS score was 6 (IQR = 2), a noticeable postoperative improvement indicated by the last follow-up score 1 (IQR = 1). Compared with the preoperative VAS scores in the UPSF and BPSF groups, the postoperative VAS scores were significantly less (*P* < 0.001) while there was no statistically significant difference between them (*P* > 0.05). Before surgery, patients in the UPSF group had an ODI 44.5 (IQR = 5), which significantly decreased after surgery with a final follow-up 14.5 (IQR = 3). By contrast, in the BPSF group, the preoperative ODI was 44 (IQR = 4) and the last follow-up ODI was 16 (IQR = 5). Collectively, both groups exhibited better postoperative functional recovery compared to pre-surgery (*P* < 0.001) while no significant difference was detected between these two groups (*P* > 0 0.05, Table 4).

**Table 4:** Comparison of VAS and ODI scores between two groups before and after surgery.

	Before operation	3 months post-operation	Last follow-up	*P* value
*VAS*				
BPSF	6 (IQR = 2)	2 (IQR = 1)	1 (IQR = 1)	*< 0.001*
UPSF	6 (IQR = 1)	2 (IQR = 1)	1 (IQR = 1)	*< 0.001*
*ODI*				
BPSF	44 (IQR = 4)	18 (IQR = 4)	16 (IQR = 5)	*< 0.001*
UPSF	44.5 (IQR = 5)	16 (IQR = 4.75)	14.5 (IQR = 3)	*< 0.001*

*UPSF* unilateral pedicle screw fixation, *BPSF* bilateral pedicle screw fixation, *ODI* oswestry disability index, *VAS* visual analog scale, *IQR* interquartile range.

### Radiological results

The analysis of postoperative radiological changes revealed that the LL angle in the BPSF group was 34.57 ± 13.32 preoperatively, which increased to 41.90 ± 10.17 at 3 months postoperatively and remained stable at 40.67 ± 9.17 at the last follow-up. In the UPSF group, the LL was 35.96 ± 9.54 preoperatively, which increased to 41.75 ± 11.57 at 3 months postoperatively and remained stable at 40.77 ± 11.97 at the last follow-up. The increase in physiological curvature of the lumbar spine after surgery showed significant differences between both groups (*P* = 0 0.007, *P* = 0.025), while there were no significant differences between the UPSF and BPSF groups at preoperative and postoperative follow-up time points (*P* > 0.05). Due to some patients in the UPSF group undergoing simple PLF without treatment of intervertebral disc space, these patients were excluded from statistical analysis regarding IDH. In the UPSF group, IDH increased from 8.21 ± 2.46 mm to 10.28 ± 2.00 mm at 3 months, and the last follow-up was 9.90 ± 1.64 mm. Similarly, in the BPSF group, the IDH increased from a preoperative value of 7.99 ± 2.18 mm to 10.33 ± 2.03 mm at 3 months after surgery and remained stable at 10.00 ± 1.63 mm at the last follow-up. Both groups exhibited improved recovery of IDH after surgery compared to before surgery (*P* < 0.001, Table 5). At the final follow-up, proximal cranial IDH decreased to (0.73 ± 0.81) mm in the UPSF group and (2.05 ± 1.59) mm in the BPSF group. The decrease observed in the UPSF group was less than that in the BPSF group with a statistically significant difference between both groups at 3-month post-operative follow-up as well as final follow-up (*P* < 0.001, Table 5).

**Table 5:** Comparison of radiological data between two groups.

	Before operation	3 months post-operation	Last follow-up	t value	*P* value
*LL*					
BPSF	34.57 ± 13.32	41.90 ± 10.17	40.67 ± 9.17	− 2.774	*0.007*
UPSF	35.96 ± 9.54	41.75 ± 11.57	40.77 ± 11.97	− 2.270	*0.025*
*IDH (mm)*					
BPSF	7.99 ± 2.18	10.33 ± 2.03	10.00 ± 1.63	− 5.436	*< 0.001*
UPSF	8.21 ± 2.46	10.28 ± 2.00	9.90 ± 1.64	− 3.747	*< 0.001*
*Decrease of IDH at cranial adjacent segmental (mm)*					
BPSF		0.99 ± 1.17	2.05 ± 1.59	–	–
UPSF		0.43 ± 0.57	0.73 ± 0.81	–	–
t value		− 3.108	− 5.369		
*P* value		*0.002*	*< 0.001*		

*UPSF* unilateral pedicle screw fixation, *BPSF* bilateral pedicle screw fixation, *LL* lumbar lordosis, *IDH* intervertebral disc height.

### Postoperative complications

In the UPSF group, one case experienced a dural tear resulting in cerebrospinal fluid leakage, and another case suffered from a postoperative epidural hematoma. Cerebrospinal fluid leakage caused by dural rupture occurred in two patients in the BPSF group, and no infections were observed in any of the surgeries. In cases of intraoperative dural rupture, it is recommended to meticulously suture the fascia layer and maintain drainage for 3–5 days post-surgery. Additionally, vigilant monitoring of drainage fluid color and volume is imperative.

## Discussion

The process of pedicle screw fixation and interbody fusion is an established technique for treating degenerative lumbar spine disease^4^ providing immediate and robust stability to the lumbar spine through BPSF while preserving the posterior ligament complex. Intraoperatively, manipulation of only one side of the dura mater reduces the risk of complications related to the dural injury. However, this approach requires the dissection of bilateral muscles and soft tissues, resulting in increased damage to these structures. Moreover, prolonged operation time increases muscle compression ischemia duration, elevating the risks of postoperative muscle atrophy^5^ perioperative infection, and intraoperative blood loss. UPSF involves the removal of only affected-side muscles while preserving contralateral tissue structure integrity and continuity of the posterior ligament complex. This mitigates low back weakness syndrome caused by denervation atrophy of healthy paravertebral muscles as well as postoperative scarring-induced spinal canal restenosis and adhesion formation while maximizing spinal stability^23–25^. Additionally, McAfee et al.^26^ study on osteoporosis associated with internal fixation concluded that excessive internal fixation could induce stress-shielding effects leading to osteoporosis in fused segments. However, Goel et al.^7^ findings demonstrated that UPSF reduces stress shielding compared to BPSF. In our study, the implants used did contribute significantly to the cost reduction in the UPSF group compared to traditional spinal fixation methods. The UPSF group only requires three pedicle screws and one rod, along with one or two interbody fusion cages. Additionally, this approach is associated with minimal blood loss, obviating the necessity for blood transfusion. On average, the operative time in the UPSF group was shorter than that in the BPSF group, which reduced the direct costs. Patients in the UPSF group generally had a shorter hospital stay. This led to less expenses including hospital room fees, nursing care costs, and the cost of routine postoperative medications. Several studies^9,15,19,20,27–30^ have reported similar clinical outcomes between UPSF and BPSF groups but highlighted advantages such as reduced operation time, intraoperative blood loss rates associated with muscle and soft tissue injuries along with lower costs associated with internal fixations in the UPSF group. In this study, no statistically significant difference was observed in the improvement of VAS leg score and ODI for lumbar and leg pain during follow-up. Furthermore, comparable efficacy was achieved in the treatment of BPSF compared to UPSF for lumbar two-level degenerative diseases.

Studies have demonstrated that UPSF in multi-level lumbar fixation involving two or more levels does not provide sufficient stability and carries a high risk of internal fixation failure, resulting in decreased fusion rates. Therefore, it is not recommended for the treatment of multi-level lumbar degenerative diseases. Yucesoy et al.^31^ conducted biomechanical simulation experiments comparing UPSF with BPSF and found that UPSF with two segments did not achieve adequate biomechanical effects. However, their study did not consider the supportive role of the anterior fusion apparatus and eliminated the protective effect of muscle tissue from the specimens, thereby increasing spinal instability. Our research group^32^ reported significantly higher axial rotation and lateral curvature in UPSF without interbody fusion device support compared to BPSF with or without an interbody fusion device at two-level fixations based on simulated biomechanical experiments on cadavers. Nevertheless, when combining UPSF with interbody fusion device implantation, there was no significant difference in terms of axial range of motion and neutral zone of the spine between BPSF with or without an interbody fusion device. This finding confirms that interbody support provides immediate stability. Gu et al.^15^ through a prospective comparative study using minimally invasive transforaminal lumbar interbody fusion (MIS-TLIF), reported 94.3% and 94.9% fusion rates for UPSF and BPSF respectively. Xue et al.^20^ concluded that the fusion rates in the UPSF and BPSF two-segment groups were 87.5% and 91.2%, respectively, with only 8 cases reported in the UPSF two-segment group. However, Suk et al.^18^ found that the fusion rate of the UPSF group (91.5%) was lower than that of the BPSF group (97.5%) in their study on cases with mixed single and double segments. Nevertheless, no detailed description was provided for the fusion rate of the UPSF group with two segments. In this study, we categorized the UPSF group into three categories based on different fusion methods: IF group (96.43%), PLF group (82.35%), and IF & PLF group (100%). The BPSF group had respective fusion rates of 97.67%, 100%, and 100%. Importantly, there were no significant differences observed in terms of fusion rates among all groups examined in this study’s findings suggest that unilateral fixation can be considered as a viable treatment option for two-level lumbar degenerative disease.

Suk et al.^18^ reported that the incidence of internal fixation failure was 12.8% in patients undergoing unilateral fixation, which was higher than the rate of 5.0% observed in those receiving BPSF and reached 30.8% among individuals diagnosed with spondylolisthesis prior to surgery. Therefore, it is recommended to avoid employing UPSF in patients with a preoperative diagnosis of spondylolisthesis. However, the specific details regarding internal fixation failure, such as screw fracture or loosening, were not provided by the authors. Among the UPSF group, 40% (4 cases) occurred in patients with single-level lumbar spondylolisthesis I accompanied by LSS or LDH before surgery; while 33% (14 cases) occurred in patients with LDH or LSS without any significant difference between screw loosening and preoperative diagnosis.

Hiyama et al.^33^ reported that a more pronounced cage subsidence rate was detected in the unilateral fixation group compared to the bilateral fixation group within the context of Lateral Lumbar Interbody Fusion (LLIF). Notably, in one particular case, the cage prolapsed, ultimately necessitating a revision surgery. While they acknowledged that, in the short-term, UPSF has the advantage of minimizing surgical-induced muscle damage and can yield comparable clinical outcomes to BPSF, it’s crucial to note that LLIF surgery hinges on the cage’s distraction mechanism to achieve indirect nerve decompression. However, UPSF fails to offer adequate support. As the cage subsidence gradually advances, there is a distinct risk of the recurrence of neurological symptoms. This concern ultimately led to the premature termination of their study. In contrast, Posterior Lumbar Interbody Fusion (PLIF) involves direct decompression of the nerves. The posterior fixation in PLIF serves to provide immediate stability, and once the fixed spinal segments have fully fused, the spine attains sufficient stability. Given these considerations, long-term follow-up is of utmost importance. This follow-up should comprehensively monitor the condition of the internal fixation, the cage, spinal stability, neurological function, and overall patient functionality.

Currently, it is widely acknowledged that a minimum X-ray clear area width of 1 mm at the bone-screw interface serves as the diagnostic criterion for screw loosening^34^. In this study, screw loosening was observed in 20 cases (48.4%) within the UPSF group, with 4 cases being cephalic, 13 cases caudal, and 3 cases involving both cephalic and caudal regions. Within the BPSF group, there were 7 cases of screw loosening, 3 cephalic, 3 caudal and 1 case exhibiting simultaneous involvement of both cranial and caudal regions. Importantly, no instances of screw loosening occurred in the middle vertebral body among all patients. The incidence rate of screw loosening in the UPSF group was significantly higher than that in the BPSF group, with approximately 65% of occurrences located at the caudal end. In contrast, within the BPSF group, screws on either side exhibited similar rates of loosening: 3 cases (42.86%) involved tail-side screws while another 3 cases (42.86%) affected head-side screws. Wu et al.^34^ discovered that lumbar internal fixation procedures involving more than two levels did not result in any instances of screw loosening within middle vertebrae; instead, cranial and caudal screws accounted for approximately 28% and 72%, respectively, with tail screws posing a higher risk for looseness or extraction. Gong et al.^35^ pointed out that in the finite element model of UPSF and BPSF of lumbar double segments, the stress on pedicle screws was primarily concentrated at the distal end of the lower screw and at the junction between the connecting rod and vertebral body. Prolonged non-fusion and excessive exposure of pedicle screws outside the bone can increase the risk of fracture. However, this study only observed screw loosening without any instances of screw breakage. In this study, IF resulted in 39.29% (11/28) cases of screw loosening, while PLF resulted in 52.94% (9/17) cases within different fusion methods in UPSF group respectively. It can be concluded that PLF is more prone to screw loosening, with simultaneous loosening observed at both ends for all 3 cases using this fusion method. Chen et al.^36^ and He et al.^22^ finite element model and biomechanical study revealed that UPSF without interbody fusion device implantation experience greater stress compared to when a fusion device is implanted, as it effectively distributes stress away from pedicle screws thereby reducing the risk of internal fixation loosening and fracture. Zhang et al.^21^ and Chen et al.^37^ have also highlighted that UPSF combined with reliable IF can be utilized for lumbar two-level degenerative disease, resulting in strong fusion and favorable clinical outcomes. IF not only enhances the fusion effect but also reduces stress on the pedicle screws. Despite the occurrence of screw loosening in the study, there were no significant differences observed in clinical outcomes compared to patients without screw loosening. Currently, based on the available follow-up data, we have not found clear evidence indicating that this screw loosening directly leads to long-term spine instability or a significant decline in patient functionality. Generally, if the fixed segments have achieved good fusion, the overall stability of the spine is reliable. We will closely monitor the long-term radiological and clinical data of the patients included in this study, such as X-ray images in flexion and extension positions and ODI (Oswestry Disability Index), in order to detect any potential trends in spine stability and patient-reported functional outcomes over time.

The findings from numerous clinical studies consistently demonstrate that UPSF is an effective approach for delaying the process of ASD during long-term follow-up periods^18,38,39^. Biomechanical studies^30^ indicate that UPSF, compared to BPSF reduces biomechanical load on the adjacent segment of the opposite side during torsion and bending activities, thereby decreasing the incidence of ASD. Intraoperative BPSF increases the likelihood of joint and nerve injuries to the upper articular process, elevates biomechanical stress on adjacent segments, and raises the risk of ASD^5,38^. A 10-year follow-up study conducted by Kim et al.^38^comparing UPSF and BPSF combined with PLF, reveals superior clinical outcomes and a lower occurrence of ASD in imaging for the UPSF group, particularly at the second cephalad segment. Chen et al.^39^ research discovered significantly lower IDH and lordosis angle in the proximal segment above after their last follow-up examination when compared with those observed before treatment within both groups. However, these differences were more pronounced in patients who underwent BPSF. Our study found a statistically significant decrease in IDH at adjacent levels among patients who received BPSF compared to those who underwent UPSF. These results suggest that while achieving comparable treatment efficacy for two-level lumbar degenerative diseases, UPSF can effectively delay the process of ASD.

Our study has some limitations. First, this study is a retrospective study. Second, the number of included cases is limited, and a longer follow-up period is required to compare the clinical effect in long-term studies. Third, further research is needed to investigate screw loosening and select more robust screws such as bone cement screws. At last, the inclusion of patients with different fusion techniques is likely a limitation, but this limitation may be minor because the fusion techniques did not make a difference between the two groups compared. Previous studies^38,40^ have shown that diverse lumbar fusion techniques can achieve satisfactory clinical outcomes in degenerative disc diseases, which is in line with our findings in the current study.

## Conclusion

The clinical efficacy of posterior unilateral and bilateral fixation in treating lumbar two-level degenerative disease is comparable, with the added benefits of unilateral fixation including reduced muscle damage, shorter operative time, decreased intraoperative blood loss and treatment costs, as well as delayed degeneration of adjacent intervertebral discs. However, it should be noted that pedicle screw loosening was more frequently observed in the unilateral fixation group compared to the bilateral fixation group.

## Data Availability

The datasets used during the current study available from the corresponding author on reasonable request.

## Abbreviations

ANOVA: Analysis of variance
ASD: Adjacent segment degeneration
BPSF: Bilateral pedicle screw fixation
BMI: Body mass index
IDH: Intervertebral disc height
IF: Intervertebral fusion
IQR: Interquartile range
LDH: Lumbar disc herniation
LL: Lumbar lordosis
LSS: Lumbar spinal stenosis
ODI: Oswestry disability index
PLF: Posterolateral fusion
TLIF: Transforaminal lumbar interbody fusion
UPSF: Unilateral pedicle screw fixation
VAS: Visual analog scale
